# Supplementary material for: Mutations in PCYT2 disrupt etherlipid biosynthesis and cause a complex hereditary spastic paraplegia
Source: Brain. 2019 Oct 22;142(11):3382–97. doi: 10.1093/brain/awz291 (PMC6821184; doi:10.1093/brain/awz291)
Supplement: awz291_Supplementary_Data [file awz291_supplementary_data.zip › awz291-Suppl_data/Supplementary_Data4.zip!/Supplementary information 3 - fibroblast lipidomics.zip!/Fibroblast lipidomics/Controle vs Patient2/Statistical plots and data/Hex2Cer[d] species/Boxplots all.pdf]

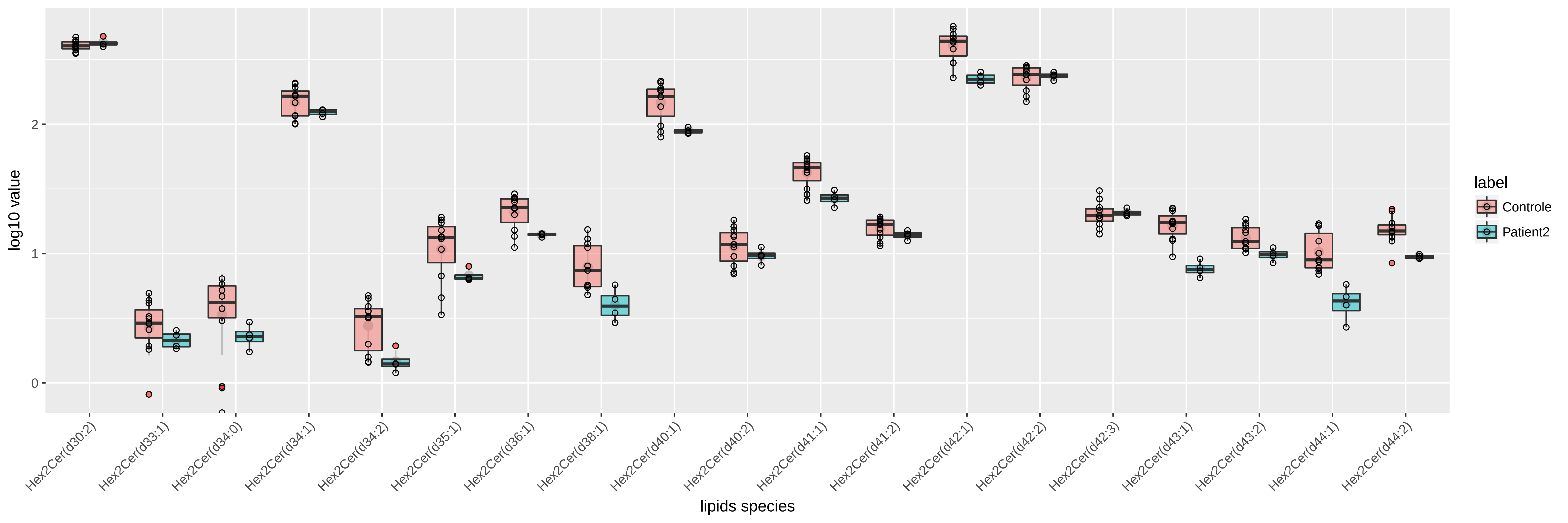
log10 value
0
1
2
Hex2Cer(d30:2)
Hex2Cer(d33:1)
Hex2Cer(d34:0)
Hex2Cer(d34:1)
Hex2Cer(d34:2)
Hex2Cer(d35:1)
Hex2Cer(d36:1)
Hex2Cer(d38:1)
Hex2Cer(d40:1)
Hex2Cer(d40:2)
Hex2Cer(d41:1)
Hex2Cer(d41:2)
Hex2Cer(d42:1)
Hex2Cer(d42:2)
Hex2Cer(d42:3)
Hex2Cer(d43:1)
Hex2Cer(d43:2)
Hex2Cer(d44:1)
Hex2Cer(d44:2)
lipids species
label
Controle
Patient2
